# Cornea Oculomics: A Clinical Blueprint for Extending Corneal Diagnostics and Artificial Intelligence in Systemic Health Insights

**DOI:** 10.3390/diagnostics15050643

**Published:** 2025-03-06

**Authors:** Ryung Lee, Rahul Kumar, Alex Weaver, Ji Hyun Kim, Arriyan Raza, Joshua Ong, Ethan Waisberg, Rahul Pandit

**Affiliations:** 1 Touro College of Osteopathic Medicine, New York, NY 10027, USA; 2 Department of Biochemistry and Molecular Biology, University of Miami Miller School of Medicine, Miami, FL 33136, USA; rahulca13@gmail.com; 3 Department of Ophthalmology, University of Florida, Jacksonville, FL 32209, USA; alex.weaver@jax.ufl.edu; 4 School of Medicine, New York Medical College, Valhalla, NY 10595, USA; celinejihyunkim@gmail.com; 5 Department of Computer Science, University of Michigan, Ann Arbor, MI 48109, USA; aaraza@umich.edu; 6 Department of Ophthalmology and Visual Sciences, University of Michigan Kellogg Eye Center, Ann Arbor, MI 48105, USA; ongjo@med.umich.edu; 7 Department of Clinical Neuroscience, University of Cambridge, Cambridge CB2 0QQ, UK; ethanwaisberg@gmail.com; 8 Department of Ophthalmology, Blanton Eye Institute, Houston Methodist Hospital, Houston, TX 77030, USA; rtpandit@houstonmethodist.org; 9 The Houston Methodist Research Institute, Houston Methodist Hospital, Houston, TX 77030, USA; 10 Department of Ophthalmology, University of Texas Medical Branch, Galveston, TX 77555, USA

**Keywords:** cornea, oculomics, medicine, artificial intelligence, biomarkers

## Abstract

Oculomics is an emerging field that leverages ophthalmic imaging data to identify biomarkers of systemic disease, facilitating early diagnosis and risk stratification. Despite its growing recognition, gaps remain in the literature regarding the clinical applications of oculomics. Various systemic diseases—including metabolic disorders (e.g., diabetes mellitus), infectious diseases (e.g., COVID-19), neurodegenerative diseases (e.g., dementia), hematologic disorders (e.g., thalassemia), autoimmune conditions (e.g., rheumatoid arthritis), and genetic syndromes (e.g., Fabry disease)—exhibit ocular manifestations detectable through in vivo confocal microscopy and anterior segment optical coherence tomography, among other imaging modalities. Increasing evidence supports the role of corneal imaging in identifying systemic disease biomarkers, a process further enhanced by artificial intelligence-driven analyses. This review synthesizes the current findings on corneal biomarkers of systemic disease, their ophthalmic imaging correlates, and the expanding role of corneal oculomics in translational medicine. Additionally, we explore future directions for integrating oculomics into clinical practice and biomedical research.

## 1. Introduction

Extending diagnostic modalities beyond their intended use may give us novel ways of managing systemic disease. One emerging field utilizes the oculome or macroscopic, microscopic, and molecular ophthalmic features associated with health and disease [1]. Oculomics then serves as a window into our systemic health [2]. Retinal imaging has demonstrated high accuracy in detecting cardiovascular, neurodegenerative, and renal diseases when integrated with artificial intelligence, through deep learning algorithms [3,4,5]. Deep learning algorithms, particularly convolutional neuronal networks, are highly applicable to ophthalmology, as these are image-based systems [6]. Key advantages of oculomics include widespread availability, non-invasive nature, and low-cost effectiveness of ophthalmic imaging modalities [7]. Despite these advantages, gaps persist in the medical literature describing the successful clinical implementation of oculomics [8]. With the increasing global burden of systemic diseases, the field of oculomics has excellent potential to be a boon to medical diagnostics [9]. Consequently, numerous funding initiatives have been established to support the advancement of this field [10].

The application of oculomics principles to anterior segment structures holds significant unharnessed potential. The anterior segment is anatomically complex and exhibits numerous associations with systemic diseases. For example, conjunctival pallor is a well-established physical examination finding indicative of anemia [11]. Similarly, scleral icterus, characterized by the yellow discoloration of the conjunctiva, serves as a recognized diagnostic indicator of hepatobiliary disorders [12]. Moreover, alterations in pupil size and ocular motility may serve as diagnostic markers for mild traumatic brain injury [13]. The comprehensive application of oculomics to all anterior segment structures could substantially expand the spectrum of systemic diseases detectable through ophthalmic imaging [14]. In addition, anterior segment diagnostic techniques, including external ocular imaging, are considerably less invasive than posterior segment assessments.

The cornea is a transparent, dome-shaped structure located at the anterior-most part of the eye, and current imaging techniques offer potential avenues for identifying systemic biomarkers. The cornea comprises five distinct layers: the epithelium, Bowman’s layer, the stroma, Descemet’s membrane, and the endothelium. The cornea responds to changes in the immune system [15]. Several systemic diseases, particularly autoimmune and metabolic disorders such as rheumatoid arthritis and diabetes mellitus, manifest in characteristic corneal findings [16]. In vivo confocal microscopy and anterior segment optical coherence tomography (AS-OCT) facilitates the detection of corneal manifestations of systemic diseases, offering promising tools for oculomics-based predictive modeling [17,18]. This review aims to synthesize current oculomics research on the relationship between anterior segment findings and potential systemic biomarkers. This review establishes a foundation for future research in anterior segment oculomics by consolidating the existing evidence.

## 2. Materials and Methods

Our search was based on previous studies and contained no new studies with human participants or animals performed by the authors. Using the medical literature databases Embase, Google Scholar, Web of Science, Grey Literature, and PubMed, we utilized the search terms related to “systemic diseases”; “early diagnosis”; “standard diagnostics”; “traditional methods”; “diagnostic accuracy”; “clinical utility”; “sensitivity and specificity”; “predictive biomarkers” AND “corneal imaging”; “anterior segment”; “oculomics”; and “artificial intelligence in ophthalmology” in search of journal articles related to our initial inquiry. Boolean operators were used to enhance the search logic, with “OR” applied to capture synonyms and related concepts, such as (“oculomics” OR “ocular biomarkers” OR “cornea systemic diagnostics”), and “AND” to combine terms for intersecting topics, such as (“cardiovascular health” OR “neurodegenerative diseases”).

We limited the search criteria from inception to October 2024. The main inclusion criteria were oculomics, systemic biomarkers, parameters, artificial intelligence, and diagnostics. Articles were selected based on article titles and abstracts. Duplicates were removed manually. Full texts were read and were further reviewed for relevance. Articles were excluded if they were not in English or were irrelevant to the cornea. These terms were searched independently or with adjacent topics using titles and abstracts. Additional articles were supplemented if the articles from search results were inconclusive. The limitations of our review methodology include selection biases in Grey Literature.

## 3. Results

We found 1273 articles from the search strategy above. A total of 217 articles were relevant to our study. We excluded 90 articles because they (1) did not meet the search criteria of “relevance to the cornea” (e.g., studies focusing exclusively on retinal or systemic conditions without corneal involvement) or (2) were duplicate results. We included the remaining 117 articles in our literature review. They provided valuable insights into corneal oculomics, biomechanical properties, topography, densitometric changes, and their relation to systemic diseases.

## 4. Discussion

### 4.1. Cornea Oculomics

#### 4.1.1. Endocrine and Metabolic Diseases

**Figure 1:**
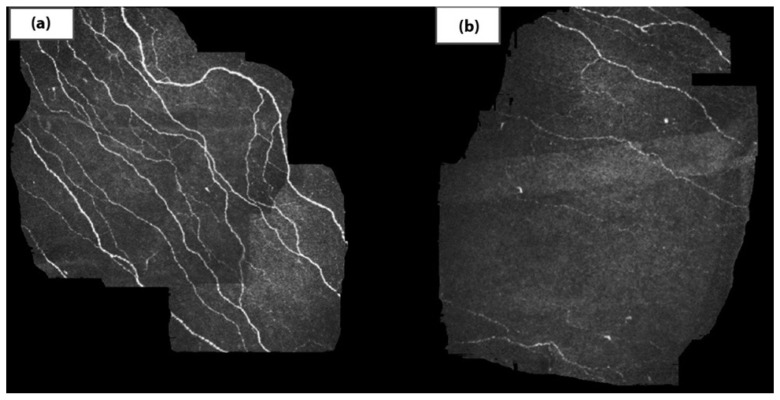
Corneal confocal microscopy showing sub-basal nerve plexus in (**a**) standard structure corneal nerve fibers and (**b**) the loss of corneal nerve fibers in type 2 diabetes. Available online: https://pmc.ncbi.nlm.nih.gov/articles/PMC4511296/ (accessed on 11 February 2025). This figure is licensed under the Creative Commons Attribution-NonCommercial License (CC BY-NC).

The potential utility of corneal biomarkers arises from the cornea’s association with a range of endocrine disorders. Corneal complications have been reported in 45–70% of individuals with diabetes mellitus [1]. Intervention at the early stages of disease is superior to late-stage management, particularly after neuropathies. Diabetic epitheliopathy is a common manifestation of diabetic corneal disease, often resulting in recurrent erosions and persistent non-healing epithelial defects [2]. Diagnostic evaluations and oculomics algorithms can leverage slit-lamp examination findings with fluorescein staining to detect these corneal abnormalities. In diabetic corneal neuropathy, in vivo confocal microscopy reveals characteristic alterations in epithelial nerves, including a reduction in long nerve fiber bundles and the thickening of the corneal sub-basal nerve plexus [3]. Additionally, abnormal hyperreflective signals may be observed at the interface between the corneal epithelium and anterior stroma [3]. Integrating refined confocal microscopy techniques with artificial intelligence can facilitate the early detection of diabetic corneal neuropathy and cellular layer alterations, thereby establishing a robust biomarker for diabetes within the cornea (Figure 1).

Graves’ disease, an autoimmune disorder, is associated with corneal alterations. Eyelid retraction and proptosis in thyroid eye disease frequently contribute to concurrent dry eye syndrome development [4]. Beyond conventional diagnostic methods for dry eye syndrome, such as staining techniques, corneal topographic alterations have been observed in patients with Graves’ disease following strabismus surgery and orbital decompression [5]. For instance, variations have been noted in parameters such as the highest concavity, A1 velocity, and A2 time. Patients with Graves’ disease exhibit increased concavity and prolonged A2 time. These measurements, obtained using the Corvis ST, assess the cornea’s biomechanical properties, including the corneal deformation velocity during application and the time required for the second application, and may serve as unique biomarkers for the presence of Graves’ disease following strabismus surgery.

In multiple endocrine neoplasia (MEN) type 2, corneal manifestations may provide additional diagnostic insights into the disease. MEN type 2 is an autosomal dominant disorder characterized by the early onset of medullary thyroid carcinoma and pheochromocytoma [6]. Therefore, early diagnosis of the condition would be helpful. In a case study, Yin et al. utilized in vivo confocal microscopy (IVCM) to characterize a patient with a nasal conjunctival neoplasm who declined surgical biopsy [7]. IVCM reveals hyperreflective, thickened nerve plexuses and neuromas with disorganized bundles of nerves. As a result, the patient was referred to an endocrinologist for further workup of the associated neoplasm [7]. Genetic testing revealed a SOS1 gene mutation without a RET mutation, leading to a provisional diagnosis of pure mucosal neuroma syndrome, a variant of MEN2B [7]. Several other clinical studies have demonstrated the thickening of the corneal nerves in both MEN 2A and 2B [6,8,9]. Corneal imaging enhances diagnostic accuracy, particularly in cases where phenotypic features of MEN2 are subtle or in subgroups, such as with pure mucosal neuroma syndrome. Similarly, neurofibromatosis type 1 (NF1) should be considered when diagnosing patients with thickened, visible corneal nerves and conjunctival neuromas. IVCM can identify statistically significant increases in corneal nerve branching and endothelial cell density [10]. Moreover, the degree of nerve bundle disorganization may contribute to refining the unique oculomics biomarker signature.

Hyperparathyroidism is an autoimmune disease caused by the abnormal secretion of the parathyroid hormone. Hypercalcemia results in calcium deposition in Bowman’s layer, leading to band keratopathy, manifesting as white corneal deposits in the anterior stroma, seen on slit-lamp examination [11,12]. Consequently, external ocular imaging integrated with artificial intelligence algorithms may enhance the diagnostic accuracy of hyperparathyroidism [13]. Current external ocular imaging techniques have demonstrated an ability to accurately classify serum calcium levels below 8.6 mg/dL [13]. Other conditions associated with hypercalcemia, such as vitamin D toxicity, multiple myeloma, Paget’s disease of bone, sarcoidosis, and juvenile idiopathic arthritis, may also result in band keratopathy [14,15]. Early-stage band keratopathy, manifesting in the peripheral horizontal regions of the cornea, may be challenging to detect via gross examination, but could be identified using AI-based image analysis algorithms. Artificial intelligence algorithms frequently demonstrate superior accuracy in predicting diseases and identifying pathological changes [16]. Band keratopathy is one known systemic sign of hypercalcemia, but location, severity, and artificial intelligence could enhance early detection.

Anterior segment imaging holds the potential to enhance diagnostic capabilities in polycystic ovarian syndrome (PCOS). Studies by Adieyeke et al. and Puthiyedath et al. have demonstrated increased central corneal thickness in PCOS patients, as measured by ultrasound pachymetry and non-contact specular biomicroscopes [17,18]. Ozturk et al. reported significantly increased corneal densitometry values across central and peripheral regions using Schiempflug imaging in patients with PCOS [19]. The distinct pattern of increased central and peripheral corneal thickness may be characteristic of PCOS and could be leveraged as a biomarker in oculomics applications.

#### 4.1.2. Infectious Diseases

Systemic infections caused by specific pathogens can involve the cornea. Corneal oculomics may facilitate the detection of subclinical or latent manifestations of these infections. Corneal and conjunctival abnormalities have been documented in patients with COVID-19 infection [20,21]. Anterior corneal swabs demonstrated infected ocular cells through 4′,6-diamidino-2-phenylindole (DAPI) staining and microscopy [22]. SARS-CoV-2 infection exhibits corneal imaging abnormalities detectable via in vivo confocal microscopy (IVCM). Barros et al. reported reduced corneal nerve fiber density (CNFD) and corneal nerve fiber length (CNFL) from IVCM in patients recovered from COVID-19 compared to healthy controls, indicative of small fiber neuropathy [23]. Semi-automated analysis of IVCM images in a COVID-19 subgroup revealed distinct morphological alterations in the corneal sub-basal nerve plexus, accompanied by corneal cell infiltration [23]. Bitirgen et al. reported similar findings and observed decreased CNFD, corneal nerve branch density (CNBD), and CNFL in patients with “long COVID” or persistent post-acute symptoms relative to healthy controls [24]. Combining reduced CNBD and CNFL with immune cell infiltration, as detected by IVCM, may serve as a robust biomarker for post-viral neuropathic changes. Other viral, bacterial, and fungal infections may also manifest in the cornea, indicating the potential of further research into oculomics applications for a broader range of infectious etiologies [25,26,27,28,29].

#### 4.1.3. Neurological and Neuromuscular Disorders

Corneal oculomics presents an innovative method for identifying biomarkers of neurodegenerative diseases through the non-invasive evaluation of corneal nerve integrity and cellular alterations. Early detection is critical for many neurodegenerative diseases, including dementia, Parkinson’s disease (PD), multiple sclerosis, amyotrophic lateral sclerosis (ALS), and mild cognitive impairment, all of which may be more readily assessed through anterior segment imaging. A well-established association exists between Alzheimer’s disease (AD) and ocular pathologies, including increased dendritic cell density and reduced corneal sensation [30]. Corneal sensitivity serves as a potential biomarker for neurodegenerative diseases. Ornek et al. reported decreased mean corneal sensitivity values in patients with AD, MS, ALS, PD, and essential tremor (ET) [31]. Notably, the decline in corneal sensitivity is more pronounced in AD and PD compared to MS [31].

**Figure 2:**
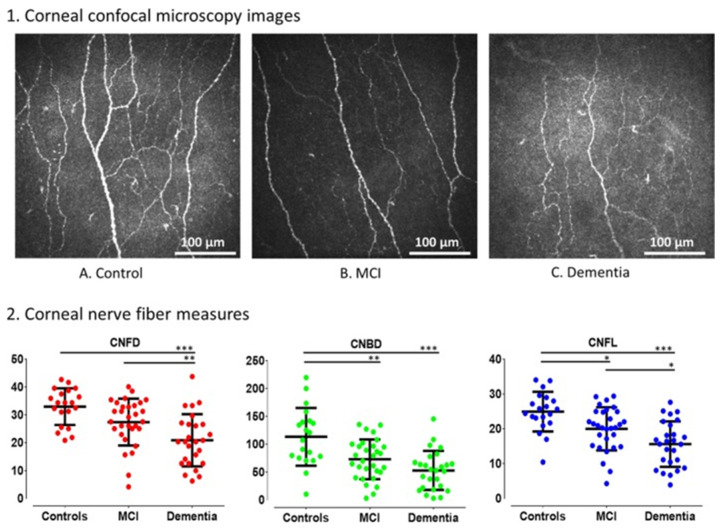
Corneal nerve fiber morphology and measurements in healthy age-matched controls, subjects with mild cognitive impairment and dementia. Graphs comparing the corneal nerve fiber density, branch density, and length are shown for the three comparison groups (* *p* ≤ 0.05, ** *p* ≤ 0.01, *** *p* < 0.0001). Available online: https://pmc.ncbi.nlm.nih.gov/articles/PMC6469344/ (accessed on 11 February 2025). This figure is licensed under the Creative Commons Attribution 4.0 International License (CC BY 4.0).

In vivo confocal microscopy (IVCM) can detect corneal nerve alterations associated with dementia (Figure 2). IVCM has demonstrated particular efficacy in early-stage dementia, revealing statistically significant reductions in corneal nerve fiber density (CNFD), corneal nerve branch density (CNBD), and corneal nerve fiber length (CNFL) during the mild cognitive impairment (MCI) stage [32,33]. Further reductions in these parameters were observed in individuals with MCI who subsequently progressed to dementia [34]. The area under the curve (AUC) for corneal nerve measurements demonstrated diagnostic accuracy comparable to that of medial temporal lobe atrophy in both MCI and dementia. The AUC values ranged from 0.78 to 0.86 versus 0.40 to 0.53, respectively [33]. IVCM offers a non-invasive alternative to magnetic resonance imaging (MRI), which is time-consuming and costly. Further advancements in measurement techniques and the integration of artificial intelligence algorithms could enhance the accuracy of neurodegenerative disease detection.

Corneal nerve measurements have the potential to identify rapid symptom progression and differentiate between patients with and without autonomic involvement in Parkinson’s disease [35]. In vivo confocal microscopy (IVCM) has demonstrated reductions in corneal nerve fiber density (CNFD), corneal nerve fiber length (CNFL), and the corneal nerve branch density-to-nerve fiber density (CNBD/CNFD) ratio in patients with symptomatic or rapidly progressive Parkinson’s disease [35,36]. IVCM has also revealed statistically significant reductions in corneal total branch density (CTBD), corneal nerve fiber area (CNFA), and corneal nerve fiber width (CNFW) in Parkinson’s disease patients [36]. Although corneal nerve measurements in dementia and Parkinson’s disease are similar, the magnitude of changes in Parkinson’s disease is generally smaller, providing a distinct corneal signature for each condition. Corneal nerve measurements can also differentiate multiple system atrophy from Parkinson’s disease [37].

IVCM can detect axonal losses in patients with multiple sclerosis. Increases in DC density correlate with the severity of inflammation in MS [38]. There are also reductions in CNFD, CNBD, and CNFL in MS patients, which correlate with an expanded disability status scale [39]. The reduction in corneal nerves parallels the retinal nerve fiber layer thinning reported across many studies [40]. Since the corneal nerves are branches of the trigeminal nerve, it makes sense that in an auto-immune mediation chronic inflammatory disease that involves the myelin sheath, there are both changes in the DC density and reductions in the corneal nerve fibers.

In a study involving patients with amyotrophic lateral sclerosis (ALS), corneal nerve fiber lengths (CNFL) were significantly reduced, whereas dendritic cell densities were markedly increased [41]. The increase in dendritic cell density was most pronounced in the inferior whorl region of the cornea [41]. ALS patients exhibited a more complex corneal nerve branch density (CNBD) distribution in the peripheral corneal regions, presenting a distinct morphological feature [41]. Overall, corneal imaging provides a foundation for the non-invasive and early detection of neurodegenerative diseases; however, further research is required to standardize imaging protocols and validate the reliability of these biomarkers.

#### 4.1.4. Autoimmune and Rheumatologic Disorders

**Figure 3:**
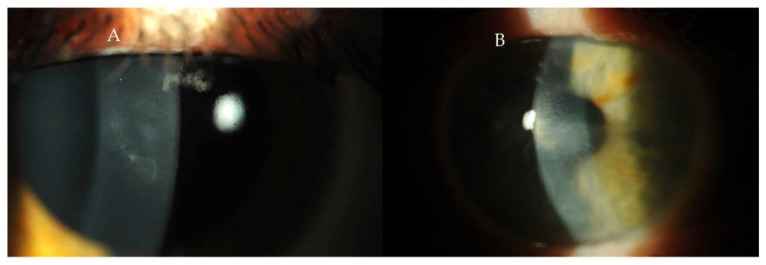
Changes in corneal topography in systemic lupus erythematosus. Available online: https://www.mdpi.com/1422-0067/23/20/12264 (accessed on 11 February 2025). This figure is licensed under the Creative Commons Attribution 4.0 International License (CC BY 4.0).

Inflammatory diseases can manifest in the cornea and be quantified using advanced corneal imaging techniques. Changes in corneal parameters, including alterations in keratometry values and corneal thickness, have been observed in rheumatoid arthritis [42]. Pentacam HR imaging has demonstrated significantly higher anterior flat (K1), steep (K2), and mean keratometry (Km) values and increased corneal thickness in patients with rheumatoid arthritis [42]. However, these findings were not replicated in a subsequent study by Ozcura et al., potentially due to a smaller sample size and the use of ultrasound pachymetry instead of Pentacam HR [43]. In such cases, integrating ophthalmic imaging findings with select laboratory tests enhances diagnostic accuracy beyond conventional diagnostic methods alone [44]. Similarly, in Sjögren’s syndrome (SS), where dry eye disease is prevalent, diagnostic assessments such as the Schirmer test for tear production, slit-lamp examination of tear break-up time, and ocular surface staining with fluorescein, rose Bengal, and Lissamine green are valuable [45,46]. In vivo confocal microscopy (IVCM) studies have shown decreased central corneal thickness (CCT) and elevated dendritic cell (DC) densities in patients with SS [47,48]. The combination of reduced CCT, increased dendritic cell density, and the decreased sub-basal nerve plexus is characteristic of Sjögren’s syndrome [47]. Systemic lupus erythematosus (SLE), a chronic inflammatory disorder, may present in a range of ocular manifestations, including keratitis, recurrent corneal erosions, peripheral corneal infiltration, dry eye disease, and cataract formation (Figure 3). In SLE, alterations in corneal biomechanical properties, such as reduced corneal hysteresis, have been detected using the Reichert ocular response analyzer [49,50]. Optical coherence tomography (OCT) has further identified decreased central corneal thickness and increased peripheral corneal thickness in patients with SLE [51,52].

Gout, a metabolic disorder characterized by hyperuricemia, may also have corneal manifestations. In vivo confocal microscopy (IVCM) measurements in patients with chronic gout demonstrated significantly increased total and higher-order aberrations compared to controls [53]. Corneal biomechanical assessments revealed significantly reduced corneal hysteresis and elevated intraocular pressure (IOP) values in the gout group [53]. Additional ocular manifestations of gout include conjunctival injection and urate crystal deposition within the cornea [54]. These corneal deposits appear as acceptable, refractile yellow crystals, detectable via slit-lamp examination [54]. Collectively, these ophthalmic imaging findings provide a foundation for future oculomics research in the context of gout.

#### 4.1.5. Genetic Diseases

Genetic disorders, particularly those impacting cellular structures such as collagen, are associated with well-documented ocular manifestations. Marfan syndrome is an autosomal dominant connective tissue disorder resulting from mutations in the *FBN1* gene encoding fibrillin-1 [55]. Patients with Marfan syndrome exhibit flattened corneas and increased corneal thickness, as measured using Orbscan corneal topography [55]. Additional ocular manifestations of Marfan syndrome include a megalocornea and an increased risk of keratoconus [56]. In contrast to Marfan syndrome, Ehlers-Danlos syndrome is associated with a microcornea rather than a megalocornea [57]. Patients with Ehlers-Danlos syndrome also exhibit a higher prevalence of myopia and tear film dysfunction [57]. Villani et al. utilized in vivo confocal microscopy (IVCM) to demonstrate corneal morphological alterations in classic Ehlers-Danlos syndrome, including astigmatism and an increase in endothelial hyperreflective dots [58]. Gharbiya et al. corroborated these findings by documenting increased corneal steepness in patients with the hypermobility type of Ehlers-Danlos syndrome [59]. Additional IVCM findings in Ehlers-Danlos patients include increased anterior and posterior stromal keratocyte densities [59].

Additional well-documented corneal associations with systemic diseases are observed in rare conditions such as Wilson’s and Fabry disease. Wilson’s disease is characterized by a Kayser–Fleischer ring, a reddish–greenish circumferential band measuring 1–3 mm in width at the corneal periphery. Anterior segment optical coherence tomography (AS-OCT) has been demonstrated to aid in the early detection of Kayser–Fleischer rings [60]. AS-OCT findings include hyperreflectivity at the level of Descemet’s membrane and the visualization of characteristic colored bands on color scale imaging [60]. Fabry disease, a lysosomal storage disorder, is an x-linked genetic disease secondary to alpha-galactosidase A enzyme deficiency. Classic symptoms typically include acral pain crisis, corneal verticillata, hypertrophic cardiomyopathy, stroke, and chronic kidney disease. Cornea verticillata, a whorl-like corneal epithelial opacity, may influence corneal biomechanical properties [61]. Cankurtaran et al. reported significant alterations in corneal densitometry and biomechanical indices—including A1 velocity, A2 velocity, deformation amplitude ratio, Corvis biomechanical index, and tomographic index—in patients with Fabry disease [62]. Furthermore, in vivo confocal microscopy (IVCM) revealed morphological changes in corneal nerves and endothelial cells in Fabry disease, including reductions in corneal nerve fiber density (CNFD) and length (CNFL), alongside increased dendritic cell (DC) density [24]. Although early ophthalmic associations of these conditions have been established within the oculome, incorporating advanced imaging modalities may yield more precise and preventive biomarkers.

A variety of genetic syndromes with corneal manifestations have been documented in the literature, including Down syndrome, polycystic kidney disease, epidermolysis bullosa, Lowe syndrome, cystinosis, xeroderma pigmentosum, Dandy–Walker syndrome, and Kartagener syndrome. The utility of corneal measurements in Down syndrome is primarily associated with the increased incidence of keratoconus in this population [63]. In patients with Down syndrome, corneal topography revealed steeper keratometry values (47.35 diopters vs. 43.70 diopters), while corneal pachymetry demonstrated reduced central corneal thickness (503 μm vs. 545 μm) compared to controls [63]. In polycystic kidney disease, the ocular response analyzer (ORA)—which evaluates corneal viscoelastic properties during bidirectional deformation induced by an air puff—demonstrated increased corneal hysteresis values without significant changes in intraocular pressure (IOP) or central corneal thickness (CCT) relative to controls [64]. While other genetic conditions exhibit corneal manifestations, definitive structural or biomechanical changes are not consistently detectable using the current ophthalmic imaging modalities.

#### 4.1.6. Hematologic and Oncologic Disorders

Corneal manifestations may offer a novel, non-invasive diagnostic avenue for patients with hematological disorders. Bouazza et al. reported that patients with acute myeloid leukemia, acute lymphocytic leukemia, multiple myeloma, and non-Hodgkin lymphoma exhibited corneal ulcers identified through slit-lamp examination and corneal imaging of the anterior segment [65]. In this cohort, 53.26% of all ophthalmic findings across various hematological disorders were localized to the anterior segment [65]. Additionally, slit-lamp examinations of patients who had undergone bone marrow transplantation revealed superficial punctate keratopathy associated with dry eye disease [66,67]. However, it remains unclear whether these anterior segment manifestations are direct consequences of the hematological disorders or secondary effects of therapeutic interventions. Chemotherapy and irradiation are known to induce ocular complications such as dry eye, corneal ulcers, and conjunctival hemorrhages [68]. In contrast, thalassemia induces anterior segment manifestations through distinct pathophysiological mechanisms that may be detectable via oculomics. For example, patients with beta-thalassemia often exhibit exocrine and endocrine abnormalities, including iron deposition in the meibomian and lacrimal glands [69]. These changes may manifest as reduced tear break-up time, decreased corneal epithelial thickness, and altered map standard deviation (MSD), which reflects the variability in epithelial thickness measurements [69]. Reductions in corneal main nerve and branch nerve densities may indicate peripheral neuropathy in beta-thalassemia patients [70]. Furthermore, patients with beta-thalassemia major often present with significantly reduced corneal topographic densitometric measurements, including decreased corneal volume and endothelial cell density, as assessed by specular microscopy [71]. A summary of corneal oculomics findings related to hematological disorders is provided in Table 1.

### 4.2. Review of Corneal Diagnostic Modalities

Anterior segment diagnostics utilize various technologies, including slit-lamp biomicroscope for qualitative assessments to advanced imaging modalities such as anterior segment optical coherence tomography (AS-OCT) and IVCM. In addition, corneal topography, which is gaining traction in oculomics, maps the surface curvature of the cornea. In contrast, corneal tomography provides comprehensive measurements of both anterior and posterior corneal curvatures and corneal thickness and volume [85]. Corneal topography can also aid in diagnosing pathological conditions associated with systemic diseases, extending beyond its traditional uses [86]. Optical coherence tomography (OCT) is a non-invasive imaging technique that employs near-infrared and low-coherence light to visualize the cornea, iris, and anterior chamber with high resolution. Integrating artificial intelligence with AS-OCT has enhanced diagnostic capabilities, facilitating tasks such as cataract grading and the precise measurement of parameters like anterior chamber depth [87,88].

In vivo confocal microscopy (IVCM) is a rapid, non-invasive ophthalmic imaging technique that permits high-resolution, cellular-level visualization of corneal microstructures [89]. IVCM is currently employed in diagnosing corneal diseases, infectious keratitis (particularly fungal and Acanthamoeba infections), and peripheral neuropathies [90]. Given the unique signature of nerve damage in these diseases, we can further define the structure of corneal oculomics. For example, the enhanced diagnosis of Acanthamoeba keratitis using confocal microscopy, where a convolutional neural network (CNN) model analyzed IVCM images and achieved a diagnostic accuracy of 76% and precision of 78% [91]. This study represented the first U.S.-based Acanthamoeba keratitis (AK) database, and the AI model demonstrated robust diagnostic performance [91]. Additionally, deep learning algorithms have been developed to improve the quantification of the corneal subbasal nerve plexus for diagnosing diabetic neuropathy, with area under the curve values of 0.83 [92,93]. Corneal densitometry, performed using Schiempflug imaging, generates tomographic maps that quantify backscattered light across various corneal regions [94]. Corneal densitometry is currently used to diagnose corneal and systemic diseases and perform prognostic evaluations following refractive surgeries [61]. A limitation of corneal densitometry is its reduced performance in evaluating corneal opacities compared to anterior segment OCT [95].

### 4.3. Applications and Future Directions in Cornea Oculomics

#### 4.3.1. Diagnostic Value of Corneal Biomarkers

In vivo confocal microscopy (IVCM) primarily facilitates the quantification of corneal nerve fiber density (CNFD), branch density (CNBD), and fiber length (CNFL), offering a lateral resolution of 1–2 μm and axial resolution of 4 μm [96]. This advanced imaging modality allows for the detailed visualization of the sub-basal nerve plexus. It is limited only by factors such as contact lens wear and existing corneal pathologies that may complicate image interpretation [38]. CNFD exhibits high diagnostic accuracy for diabetic neuropathy (AUC = 0.88; 95% CI: 0.78–0.98), frequently identifying neuropathic changes before detectable intraepidermal nerve fiber density alterations [97]. These findings have prompted the implementation of earlier neuroprotective interventions in managing diabetes [81].

Beyond nerve fiber analysis, specular microscopy offers valuable insights into endothelial cell morphometry, further enhancing ocular health assessment [98]. Specular microscopy studies have revealed that diabetic patients exhibit significantly reduced nerve fiber density (4.59 ± 0.92 versus 3.69 ± 0.44) and nerve branch density (29.05 ± 3.07 versus 20.13 ± 3.14) compared to healthy controls, indicating considerable endothelial cell damage [99]. In addition to cellular changes, corneal biomechanics assessed using devices such as the ocular response analyzer (ORA) provide insights into the viscoelastic properties of the cornea, which are influenced by collagen and proteoglycan architecture [100]. Corneal hysteresis (CH), a measure of ocular rigidity, can be influenced by various systemic conditions and connective tissue disorders [101]. For instance, patients with severe obstructive sleep apnea (OSA) demonstrate significantly reduced CH values (9.8 ± 1.4 mmHg) compared to controls (10.3 ± 1.1 mmHg; *p* < 0.001) [102]. Future research should focus on standardizing imaging protocols, developing robust normative databases across diverse populations, and evaluating the longitudinal stability of corneal biomarkers.

#### 4.3.2. Potential for Early Disease Detection

The cornea’s anatomical accessibility and physiological sensitivity renders an optimal site for early disease detection and systemic health assessment. Again, alterations in corneal nerve morphology frequently precede the onset of clinical symptoms in neurodegenerative diseases [34]. Reductions in corneal nerve fiber density (CNFD) and corneal nerve branch density (CNBD) are associated with an elevated risk of developing motor symptoms in Parkinson’s disease (Hazard Ratio [HR]: 2.1; 95% CI: 1.2–3.7) [14]. Similarly, patients with Alzheimer’s disease and mild cognitive impairment demonstrate significant reductions in corneal nerve parameters. In vivo confocal microscopy (IVCM) provides high-resolution visualization of corneal nerve structures, combining ultra-high-resolution optical coherence tomography (OCT) images and enabling comprehensive assessment of corneal and retinal changes [90]. IVCM excels in the detailed analysis of corneal nerve architecture, but is constrained by a limited field of view. In contrast, OCT provides a broader structural context, with slightly lower spatial resolution [103]. This diagnostic capability has led to the routine incorporation of corneal imaging into health screenings for individuals at high risk of systemic diseases [104]. Recently, clinicians have begun to incorporate corneal hysteresis measurements into routine examinations.

#### 4.3.3. Artificial Intelligence and Applications in Marginalized Areas

The integration of artificial intelligence (AI) with ophthalmic imaging technologies transforms diagnostics, particularly in marginalized or rural settings [105]. Generalizability across diverse populations remains critical [106]. Many existing AI training datasets are derived from predominantly Western, educated, industrialized, rich, and democratic (WEIRD) populations, which may introduce algorithmic biases and limit applicability to broader populations [107]. AI-powered diagnostic systems significantly enhance access to specialized ophthalmic care in underserved and remote regions [108].

#### 4.3.4. Challenges and Limitations

Biomarkers are heterogeneous and can be categorized as diagnostic, monitoring, or predictive, depending on their clinical application. Therefore, the initial stages may have a “trial-and-error” period. Additionally, biomarkers may not all meet surrogate endpoints for clinical research [109]. Thus, FDA-approved tests may not become available for clinical use [110]. Significant efforts are still needed to improve the automatic acquisition and quantitative analysis of imaging biomarkers [111]. Since oculomics often relies on artificial intelligence algorithms, the ethics governing artificial intelligence apply to utilizing these principles in the healthcare setting. The potential ethical implications of artificial intelligence in medicine are multiple and multidimensional. The first problem lies with the transparency and explainability of how artificial intelligence derives its conclusions [106]. This is the so-called “black box” dilemma, in which non-maleficence principles are potentially violated when the algorithm’s conclusion process is unknown [112]. Black box ethics are most pronounced in AI algorithms implementing ANNs and CNNs, due to the many non-linear interactions between the network and hidden layers. Extensive data usage and artificial intelligence are closely linked, especially in oculomics. Large numbers of images within large datasets are required to train deep learning algorithms [103,113]. Data privacy and security are significant concerns, such as during training algorithms [114]. Additionally, all AI algorithms are susceptible to adversarial attacks due to local inabilities [115]. Solutions to these problems are emerging; we can make AI algorithms more explainable, and better security measures may be developed in conjunction with the U.S. military and other government regulatory forces.

#### 4.3.5. Future Research Directions

Future research validating corneal oculomics should model previous oculomics research designs. Using large datasets that include several clinical parameters, Poplin et al. and Rim et al. developed highly accurate retinal oculomics algorithms that encompass many disease states [116,117]. External eye images relevant to the anterior segment could be used to determine systemic biomarkers, including TSH, ALT, and AST [13]. Publicly available databases of IVCM and AS-OCT imaging and health demographic variables would help international research efforts to develop corneal oculomics algorithms. However, the development of both databases and algorithms must prioritize data security, compliance with the Health Insurance Portability and Accountability Act (HIPAA) regulations, and ethical considerations. The availability of large, publicly accessible datasets would facilitate external validation and enhance the accessibility of corneal oculomics algorithms.

## 5. Conclusions

The widespread availability of diagnostic imaging modalities with robust performance metrics positions anterior segment imaging as a valuable tool for advancing our understanding and prevention of systemic diseases, including metabolic, infectious, inflammatory, autoimmune, neurodegenerative, and genetic conditions [19]. Future studies should focus on developing and validating corneal oculomics algorithms to ensure their clinical applicability and reliability. Several critical questions must be addressed to fully realize this potential: (1) What strategies can be employed to generate large-scale datasets, given that anterior segment imaging is not routinely performed? (2) How can biomarkers be effectively selected, validated, and standardized for clinical application? (3) What ethical and safety frameworks should guide the integration of artificial intelligence into anterior segment oculomics? We encourage future researchers to explore these pivotal questions and contribute to expanding this rapidly evolving field with high diagnostic and prognostic potential.

## Figures and Tables

**Table 1:** Summary of diseases detectable through corneal oculomics.

Category	Disease	Cornea Manifestations	Imaging	**Source**
Endocrine and Metabolic Diseases				
	Diabetes Mellitus	Decrease in corneal nerve fiber length Decrease in corneal nerve fiber density Increase in corneal epithelial cell density Lower sub-basal nerve density Increased corneal nerve tortuosity Increased central corneal thickness Reduced corneal optical density	IVCM Ultrasound Pachymeter	Utsunomiya et al. [72] Hashemi et al. [73] Medina et al. [74] Cuadrado et al. [75] Hossain et al. [76] Kallinikos et al. [77] Misra et al. [78] H.W. Su [79] Ramm et al. [80] Jiang et al. [81]
	Graves’	Increased highest concavity Prolonged A2 time	Corvis ST	Soleymanzadeh et al. [5]
	Multiple Endocrine Neoplasia	Hyperreflective nerve plexus Corneal nerve thickening Disorganized nerve bundle	IVCM	Yin et al. [7] Kinoshita et al. [8] Javadi et al. [9] Petrie et al. [6]
	Neurofibromatosis Type 1 Syndrome	Increased corneal nerve branching Increased corneal endothelial cell density	IVCM	Moramarco [10]
	Hyperparathyroidism	Band keratopathy	Slit-lamp examination	Golan et al. [11] Abeysiri and Sinha [12]
	Polycystic Ovarian Syndrome	Increased central and peripheral corneal densitometry Increased central corneal thickness	Pentacam Non-contact specular biomicroscope Corneal pachymetry	Ozturk et al. [19] Puthiyedath et al. [17] Adiyeke et al. [18]
Infectious Diseases				
	SARS-CoV-2	Reductions in corneal nerve fiber density, corneal nerve branch density, corneal nerve fiber length, and corneal nerve branch thickness	IVCM	Barros et al. [23]
Neurological and Neuromuscular Disorders				
	Alzheimer’s Disease	Decreased corneal sensitivity Reduction in corneal nerve fiber density Corneal nerve branch density Corneal nerve fiber length	Cochet–Bonnet esthesiometer IVCM	Ornek et al. [31] Al-Janahi et al. [33] Ponirakis et al. [32]
	Parkinson’s Disease	Reduced corneal nerve fiber density, corneal nerve branch density, corneal nerve fiber length, and CNBD/CNFD ratio Decreased corneal sensitivity	Cochet–Bonnet esthesiometer IVCM	Ornek et al. [31] Niu et al. [37] Lim et al. [36] Che et al. [35]
	Multiple Sclerosis	Decreased CNFD, CNFL, and CNBD Corneal sensitivity	Cochet–Bonnet esthesiometer IVCM	Ornek et al. [31] Mikolajczak et al. [82] Dericioglu et al. [83]
	Amyotrophic Lateral Sclerosis	Decreased CNFL Increased dendritic cell density Complex CBFD	IVCM	Fu et al. [41]
Autoimmune and Rheumatologic Disorders				
	Rheumatoid Arthritis	Increased K1, K2, and Km Decreased CCT, ACT, TCT, and CV	Pentacam HR Oculus	Ozkaya et al. [42]
	Sjogren’s Syndrome	Decreased CCT Higher dendritic cell density Patchy alterations and irregularities	IVCM	Villani et al. [47] Hao et al. [48] Tuominen et al. [84]
	Systemic Lupus Erythematosus	Lower corneal hysteresis Lower corneal resistance factor Lower CCT Higher peripheral corneal thickness	Reichert ocular response analyzer OCT Schiempflug imaging	Yazici et al. [50] Saldana-Garrido et al. [51] Eissa et al. [52]
	Gout	Increased total and higher order aberrations Lower corneal hysteresis	IVCM	Icoz et al. [53]
Genetic Diseases				
	Marfan Syndrome	Increased corneal thickness	Orbscan corneal topography system	Nehemet [55]
	Ehlers-Danlos	Thinner and steeper corneas Thinner stroma Lower keratocyte densities Increased endothelial hyperreflective dots	IVCM	Villani et al. [58] Gharbiya et al. [59]
	Wilson’s Disease	Intense hyperreflective band	AS-OCT	Sridhar [60]
	Fabry Disease	Increased corneal densitometry values A1 velocity, A2 velocity, deformation amplitude ratio, Corvis biomechanical index, tomographic and biomechanical index, and stiffness parameters Reduced corneal sensitivity Reduced corneal nerve fiber density Reduced nerve fiber length Increase in DC density	Pentacam HR Corvis ST IVCM Contact corneal esthesiometer	Cankurtaran et al. [62] Bitirgen et al. [24] Yang et al. [61]
	Down Syndrome	Increase in steepest keratometry Decrease in CCT	Corneal topography Corneal pachymetry	Alio et al. [63]
	Polycystic Kidney Disease	Increased corneal hysteresis	ORA	Serefoglu Cabuk et al. [64]
Hematological				
	Thalassemia	Decreased tear break-up time Corneal epithelial thickness Decreased branch density Corneal topographic parameters (K2, CV) Endothelial cell density	Corneal confocal microscopy Pentacam OCT Specular microscopy	Ebeid [69] Khan [70] Hanna [71]
	Leukemias (ALL, AML, and NHL, etc.)	Superficial punctate Corneal ulcers Conjunctival hemorrhage	Slit-lamp biomicroscope	Bouazza et al. [65] Hoehn et al. [67]

## Abbreviations

The following abbreviations are used in this manuscript:

ANN	Artificial neural network
AS-OCT	Anterior segment optical coherence tomography
IVCM	In vivo confocal microscopy
CH	Corneal hysteresis
CNFL	Corneal nerve fiber length
CNFD	Corneal nerve fiber density
CNBD	Corneal nerve branch density
CNBT	Corneal nerve branch thickness
CNN	Convolutional neural network
DC	Dendritic cell
MEN	Multiple endocrine neoplasia
PUK	Peripheral ulcerative keratitis
SLE	Systemic lupus erythematosus
